# Supplementary material for: Controlled Delivery of BET-PROTACs: In Vitro Evaluation of MZ1-Loaded Polymeric Antibody Conjugated Nanoparticles in Breast Cancer
Source: Pharmaceutics. 2020 Oct 19;12(10):986. doi: 10.3390/pharmaceutics12100986 (PMC7589709; doi:10.3390/pharmaceutics12100986)
Supplement: Supplementary file 1 [file pharmaceutics-12-00986-s001.pdf]

pharmaceutics

MDPI

Supplementary Materials: Controlled Delivery of BET-PROTACs: In Vitro Evaluation of MZ1-loaded Polymeric Antibody Conjugated Nanoparticles in Breast Cancer

Francisco J. Cimas, Enrique Niza, Alberto Juan, María del Mar Noblejas-López, Iván Bravo, Agustín Lara-Sanchez, Carlos Alonso-Moreno and Alberto Ocaña

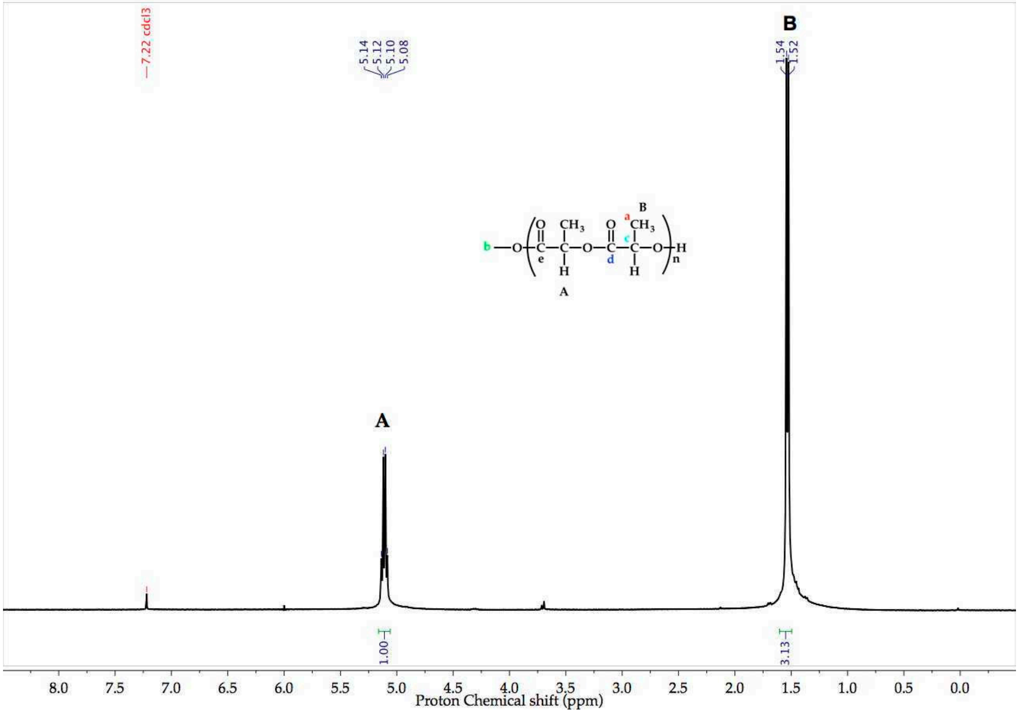

Figure S1. ^{1}H NMR of PLA.

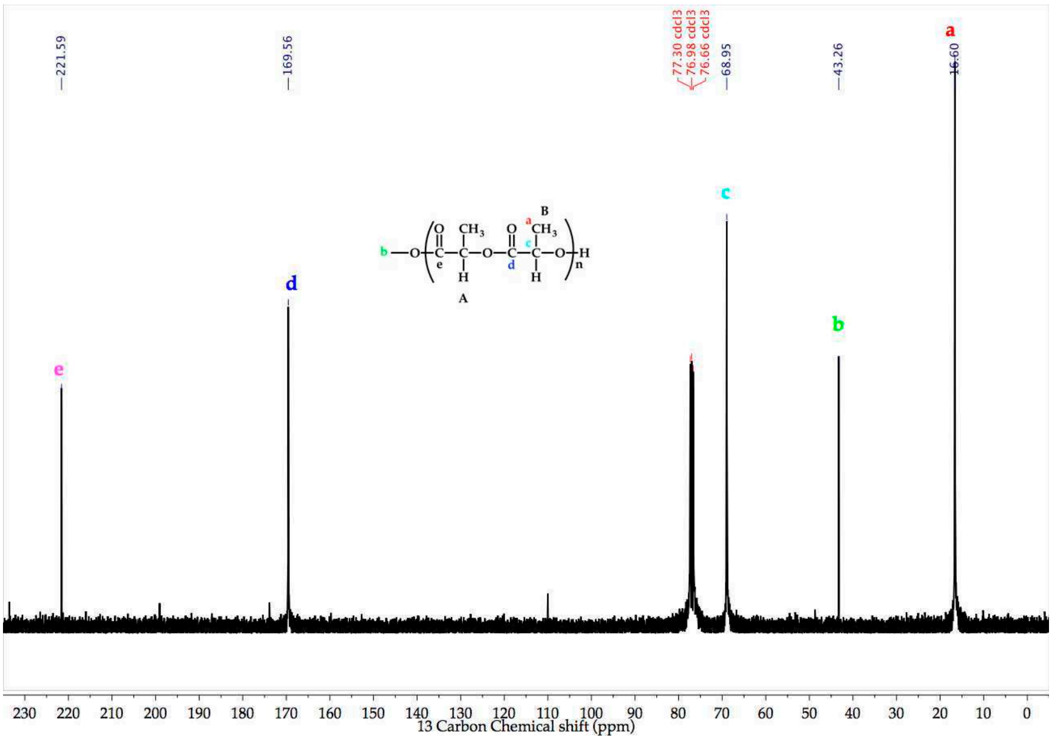

Figure S2. $^{13}C\{^{1}H\}$ NMR of PLA.

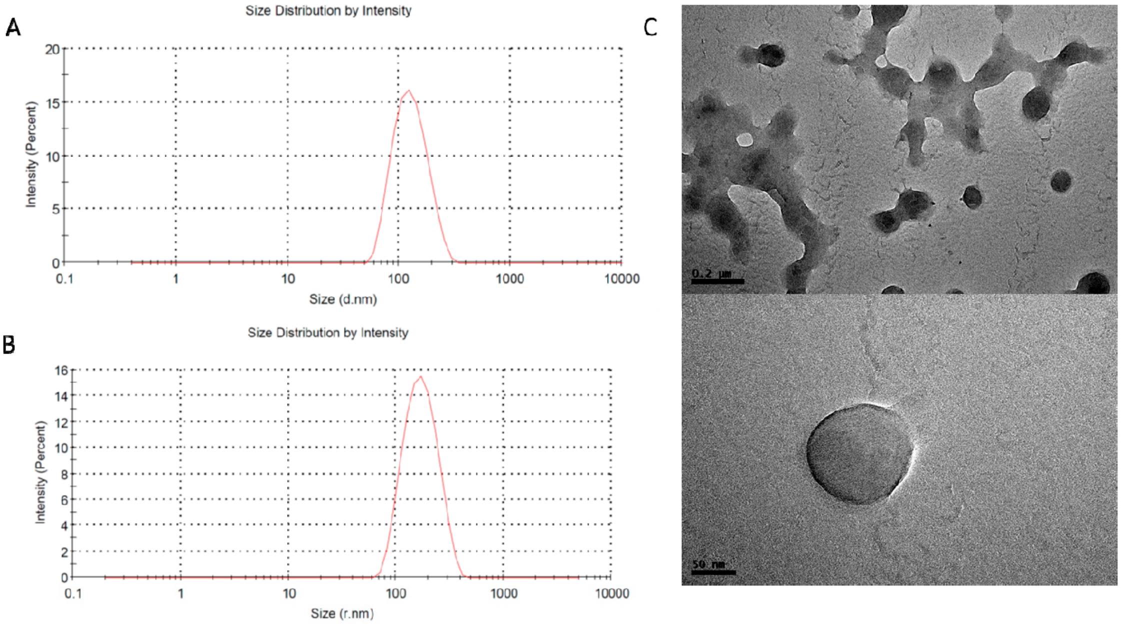

Figure S3. Size distribution curve for A) MZ1-NPs, B) MZ1-ACNPs and C) TEM images for MZ1-NPs.

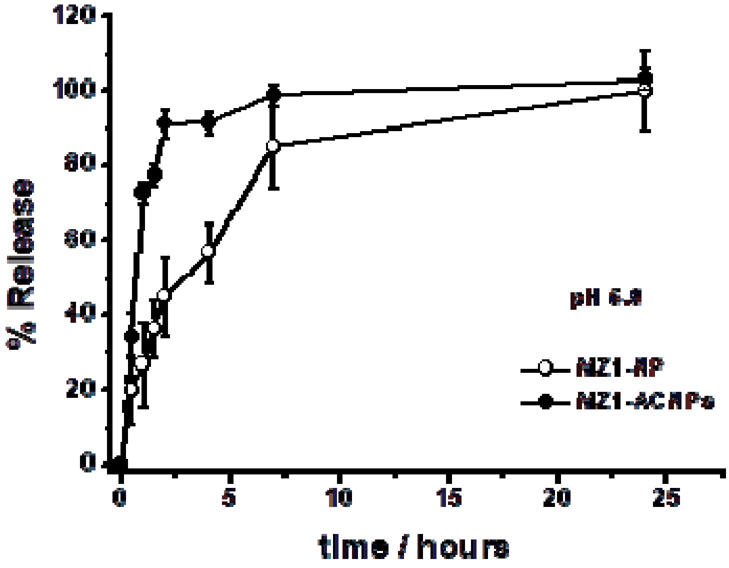

Figure S4. In vitro release profiles of MZ1-ACNPs at pH 5.8. Data are expressed as mean ± s.e.m. from at least three independent experiments.

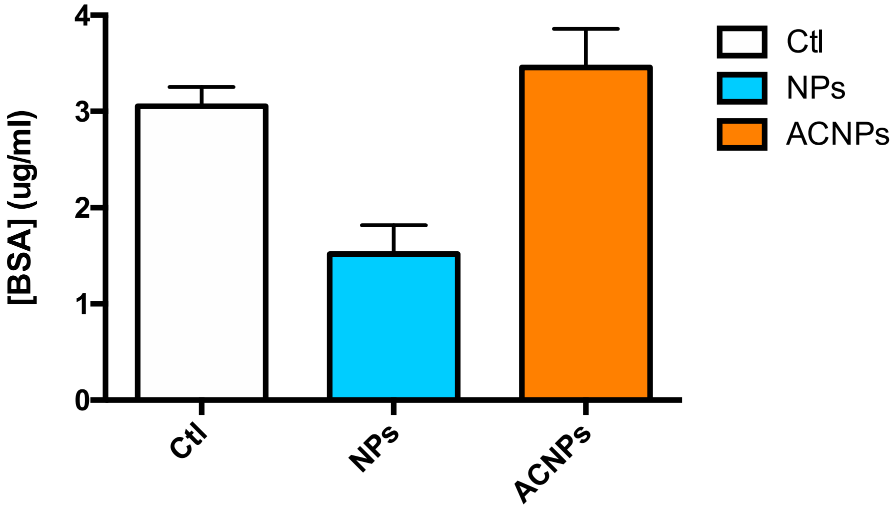

Figure 5. BCA analysis of protein corona formation. .
